# The relationship between perineal size and episiotomy during delivery

**DOI:** 10.25122/jml-2021-0390

**Published:** 2022-11

**Authors:** Nahid Radnia, Shahedeh Khansari, Nasrin Jiriaei, Seyedeh Arezoo Hosseini, Laleh Salemi, Minoo Hamoon

**Affiliations:** 1 Department of Obstetrics and Gynecology, School of Medicine, Fatemieh Hospital, Hamadan University of Medical Sciences, Hamadan, Iran; 2 Department of Social Medicine, School of Medicine, Hamadan University of Medical Sciences, Hamadan, Iran

**Keywords:** perineal size, episiotomy, perineal tear

## Abstract

Women have anatomically different perineal sizes. Different perineal sizes in primiparous women may be related to episiotomy and its consequences. The aim of this study was to investigate the relationship between perineal size and episiotomy during delivery. 376 primiparous women referred to Fatemieh Hospital in Hamadan with single pregnancies, in the first stage of labor, and with a gestational age of 37 weeks and more, out of which 372 participated in this study. Before entering the first stage of labor, they underwent perineal measurements, such as perineal body (PB), genital hiatus (GH), and anogenital area (AGD), in a forced position. Then, they were followed up for episiotomy and vaginal wall rupture until the end of the second stage of labor. Our outcome in this study was an episiotomy during delivery. The mean gestational age was 39.10±1.21 weeks, and the neonatal weight was 3107.37±42.39 g. 86.3% of women had an episiotomy, and 5.4% experienced perineal tear (laceration). Mean perineal size was 3.99±0.77, genital hiatus was 3.040±0.74, GH+PB was 7.39±1.05, and AGD was 8.49±1.22 cm. There was a statistically significant relationship between perineal body size episiotomy (P=0.011), GH+PB (P=0.003), AGD (P=0.017), neonatal birth weight (P=0.021), as well as grade 1 and 2 rupture (P<0.001). The size of GH+PB at the cut-off point of 6.25 cm and AGD at the cut-off point of 9.25 cm significantly increased the chance of performing an episiotomy. In primiparous women with a gestational age of 37 to 42 weeks, vaginal delivery, shorter perineum, vaginal hiatus, and anogenital distance significantly increased the likelihood of an episiotomy. On the other hand, performing an episiotomy significantly reduced grade 1 and 2 perineal tear rates.

## INTRODUCTION

Episiotomy is the most common intervention performed in approximately 15% to 95% of deliveries. According to recent studies, at least 80% of mothers giving birth for the first time in the United States have experienced this painful incision [1]. In Iran, episiotomy is still commonly used, and its prevalence was reported in more than 41% of primiparous women [2].

Fetal indications such as shoulder dystocia and breech delivery necessitate episiotomy to prevent perineal tears [3]. Episiotomy, like any other surgical incision, has risks, including pain, bleeding, infection, abscess, hematoma, damage to the sphincter and anal mucosa, the fistula between the anus and vagina, and painful intercourse [4]. Limiting episiotomy electively reduces its incidence by up to 30%, and thus the risk of severe perineal injury, the need for sutures, unpleasant complications such as pain, painful intercourse, urinary incontinence, and possibly anterior perineal injury [5].

According to a systematic review conducted by the World Health Organization, the scientific evidence only supports episiotomy in 5% to 20% of cases [1]. Given that there is no evidence of episiotomy prevalence, this measure should be selectively limited to specific maternal and fetal cases [6, 7].

Perineal measurements have been standardized by the International Society of Incontinence. This society defines the pelvic organ prolapse (POP-Q) quantitative measurement system as an accurate and practical technique for describing the position of pelvic organs and measuring peripheral anthropometrics. The short perineal length is associated with a high rate of episiotomy, spontaneous rupture, or instrumental delivery [8, 9]. Previous studies have been confounded by high rates of episiotomy, multiparous patients, and a retrospective design. Therefore, this study was performed to evaluate perineal measurement parameters and determine their role in the duration of the second stage of labor.

## MATERIAL AND METHODS

In this prospective cohort study, 376 primiparous pregnant women referred to Fatemieh Hospital in Hamadan for delivery were examined for perineal size, the need for episiotomy, and vaginal wall damage from April 2020 to August 2020. Assuming a 50% prevalence of episiotomy (p=0.5, q=0.5) and an accuracy of 5% (d=0.05), the sample size was estimated at 384. During that time, we only had access to 376 eligible primiparous women who entered the study. Considering the inclusion criteria, participants were selected by convenience sampling method. Inclusion criteria were willingness to participate in the study, primiparous, singleton pregnancy, gestational age 37 weeks and above, and estimated fetal weight under 4 kg.

Also, episiotomy due to fetal dystocia, fetal distress, hasty discharge, neonatal weight over 4 kg, vaginal delivery with instruments, and eligibility for emergency cesarean section were among the exclusion criteria. The objectives of the study were described for all the eligible patients at the beginning of hospitalization. Then, oral and informed consent was obtained by second-year assistants in the emergency room from those wishing to participate in the project before entering the second stage of labor. To avoid possible impact on the fetal head, the parameters were subjected to perineal measurements (PB, GH, and AGD in a forced position, measured with a graduated swap marked by a ruler). The genital size was measured from the center of the metatarsal urethra to the posterior midline of the hyenas. For perineal measurement, the distance between the posterior margin of the genital hiatus and the midline of the anus was measured. The data were then recorded in a checklist designed by the researchers.

Perineal sizes were perineum body (PB): the distance between the posterior margin of the genital hiatus and the middle part of the anus at the time of exertion; genital hiatus (GH): the distance between meatus urethra to the posterior middle line during exertion [8, 10]; anogenital size (AGD): the distance between the external genitalia from the anterior part of the clitoris to the anus and PB+GH [11]. Data collected through questionnaires were analyzed using SPSS-21. Descriptive qualitative data were expressed in tables, graphs, ratios, and percentages. Quantitative variables with normal distribution were expressed as mean and standard deviation and qualitative variables as ratio and percentage. In the analytical statistics section, Fisher's exact test was used to compare nominal qualitative variables in the group with and without episiotomy. Furthermore, Student's t-test was used to compare quantitative variables with normal distribution, and Mann-Whitney for quantitative variables abnormally distributed. All analyses were performed at a 95% confidence level.

## RESULTS

In this study, which aimed to determine the relationship between perineal size and the likelihood of requiring episiotomy during childbirth in primiparous women, 376 women were selected. 4 participants were excluded from the study due to dilatation arrest (n=1), lack of response to induction (n=1), fetal distress (n=1), and meconium (n=1).

The mean gestational age of mothers was 39.10±1.21 weeks, and the mean birth weight of their infants was 3,107.42±373.39. Out of 372 women remaining, 351 (86.3%) needed an episiotomy, while 51 (13.7%) did not. Mean perineal body size (3.99±0.77), genital hiatus (3.40±0.74), GH+PB (7.39±1.05), and AGD (8.49±1.22) are presented in Table 1. Of the 372 primiparous women, 20 (5.4%) had a ruptured vaginal wall to the sphincter or rectal mucosa, 15 patients had grade 1 rupture, and 5 patients had grade 2 rupture (Table 2). All first- and second-degree perineal tear cases were in primiparous women without episiotomy (P<0.001).

**Table 1:** Frequency distribution of perineal body size, genital hiatus, GH+PB and AGD in primiparous women.

Variable	Average	Standard Deviation	Minimum	Maximum
Perineal body (cm)	3.99	0.77	2.50	6
Genital hiatus (cm)	3.40	0.74	2.0	5.50
GH+PB (cm)	7.39	1.05	5.0	10.50
AGD (cm)	8.49	1.22	5.00	11.00

**Table 2:** Frequency distribution of grade 3 and 4 ruptures in deliveries with and without episiotomy.

Perineal tear	No	Percentage
First grade	15	4.1
Second grade	5	1.3
No rupture	352	94.6
Total	372	100

There was a statistically significant difference between primiparous women with and without episiotomy in terms of mean perineal body PB size (P=0.011), GH+PB (P=0.003), AGD (P=0.017), and weight during birth (P=0.021) (Table 3). Grouping perineal parameters, perineal body size of less than 4 cm, PB+GH of less than 6.75 cm, and AGD of less than 9.25 cm significantly increased the chance of episiotomy (Table 4). Also, in primiparous women with vaginal delivery and perineal tear, mean PB length, PB+GH, and AGD were significantly higher than those without perineal tears. However, no significant difference was observed between the two groups in terms of GH length (Table 5). The size of GH+PB at the cut-off point of 6.25 cm and AGD at the cut-off point of 9.25 cm significantly increased the chance of an episiotomy.

**Table 3:** Frequency distribution of episiotomy in primiparous women with and without episiotomy.

Variable	Episiotomy		P-value
	Yes	No	
Perineal body (cm)	3.95±0.75	4.27±0.90	0.011*
Genital hiatus (cm)	3.37±0.72	3.59±0.88	0.151*
GH+PB (cm)	7.32±1.00	7.84±1.24	0.003*
AGD (cm)	8.42±1.19	8.93±1.28	0.017*
Birth weight (week)	3125.20±357.31	2995.49±450.10	0.021**
Gestational age	39.13±1.22	38.94±1.17	0.292**

* – Mann Whitney test; ** – T-student test.

**Table 4:** Frequency distribution of episiotomy in primiparous women under vaginal delivery in terms of perineal size.

Variable	Episiotomy			OR	95% CI	Sig*
	Yes F. (%)	No F. (%)	Total F. (%)			
**Perineal body size**						
<4 cm	29 (10.5)	246 (89.5)	275 (100)	1.40	1.21–1.74	0.003
≥4 cm	22 (22.7)	75 (77.3)	97 (100)			
**PB+GH**						
<6.75 cm	5 (5.5)	86 (94.5)	91 (100)	1.30	1.12–1.77	0.009
≥6.75 cm	46 (16.4)	235 (83.6)	281 (100)			
**AGD**						
<9.25 cm	34 (11.8)	255 (88.2)	289 (100)	1.49	1.26–1.94	0.030
≥9.25 cm	17 (21.2)	63 (78.8)	80 (100)			

* – Chi-square test.

**Table 5:** Frequency distribution of perineal tear in primiparous women under vaginal delivery.

Perineal index	Perineal tear		P-value*
	Yes	No	
PB	4.42±0.85	3.97±0.76	0.018
GH	3.42±0.94	3.39±0.73	0.866
PB±GH	7.85±1.30	7.37±1.03	0.011
AGD	9.22±1.16	8.45±1.21	0.029

* – Mann-Whitney test.

## DISCUSSION

In this study, 86.3% of primiparous women underwent vaginal episiotomy, and 5.4% developed first- and second-degree perineal tears, most of whom had the former. All women without an episiotomy had the rupture of the vaginal wall to the sphincter or rectal mucosa. A recent study by Raja et al. in India on 150 primiparous women found that more than 80% needed an episiotomy, consistent with our results [12]. Our findings also showed a significant difference between smaller perineal body size, anogenital distance, total perineal body, and vaginal hiatus with increased episiotomy and decreased perineal tear.

In 2018, Moya et al. studied 119 primiparous vaginal women and found that the shorter length of GH+PB, roughly equivalent to AGD AC, was a risk factor for episiotomy. In this study, the cut-off points of >77 mm for GH+PB and >93 mm for AGD were considered risk factors for an episiotomy [13]. In the present study, the size of GH+PB at the cut-off point of 6.25 cm and AGD at the cut-off point of 9.25 cm significantly increased the chance of performing an episiotomy. The results of our study are consistent with those of Moya et al. in terms of the relationship between perineal length and episiotomy. In 2017, Farghaly et al. performed a study in Egypt on 483 women to determine the effect of perinatal length in predicting labor progression and repairable perineal tear. The mean time of the second stage of labor in women with a perineum length of 4 cm or more was significantly higher than those with less than 4 cm. The chance of vaginal rupture (OR=1.96) in women with short perineum without episiotomy was significantly higher. The researchers concluded that the risk of vaginal rupture in the short perineum is higher [14].

Consistent with Farghaly et al. [14], longer perineum length was associated with an increased duration of the second stage of labor in our study. However, because women with shorter perineum lengths underwent episiotomy, they had lower rates of vaginal rupture. In this study, women with lower PB had episiotomies. Consequently, the mean PB in women with perineum rupture was significantly higher than those without. Geller et al. studied the PB length as a risk factor for grade 1 or 2 rupture in primiparous women undergoing vaginal delivery and cesarean section (70 women with a gestation age of 35 to 37 weeks). According to the results, the short perineal body increased the risk of grade 1 or 2 rupture in primiparous women [15]. In the present study, only women undergoing genital delivery were studied. The sample size of our study was larger than that of Geller et al. [8].

In a study conducted by Rahma et al. on 47 primiparous and multiparous females, the mean length of the hiatus was 2.57±0.76 cm, and the mean length of the perineal body was 2.85. 0.91 cm. There was no significant difference between primiparous and multiparous women in hiatus and perineal body length [16]. Only primiparous women were included in our study, and their mean perineal size was 3.99 cm, while genital hiatus was 3.40 cm. In the study by Rizk et al. in the United Arab Emirates (UAE), the average length of the perineum was 4.6 cm [17], showing the difference in perineal sizes in various races. In a study by Eid SM et al. on 100 pregnant women, those with PB less than 3.5 cm had a higher rate of a perineal tear than PB above 3.5 cm. There was no significant difference in the length of GH between the two groups of episiotomy and non-episiotomy [18].

Our study observed a significant relationship between perineum rupture and lower PB. However, there was no significant relationship between the GH length and an episiotomy. Also, instead of a cut-off point of 3.5 for PB, a cut-off point of 4 cm was used. The sample size of the present study was larger than that of Eid SM et al. In the study of Dua et al. in the United Kingdom, the mean PB length per 1,000 Caucasian females was 3.7±0.9 cm, while that of Asians was 3.6±0.9 cm (2009). Primiparous women with shorter perineum (P=0.03) had significantly higher grade 3 rupture [19]. In our study, the mean PB length was about 3.9 cm, and no cases of grade 3 perineal tears were observed. Contrary to the findings of Dua et al., in our study, the size of the perineum was associated with perineal tear. The small perineal size was significantly associated with a lower probability of episiotomy. In 2000, Rizk et al. studied the relationship of perineal length with anus position and vaginal delivery in 212 primiparous women. Perineal length and anal index were measured in the first stage of labor. The mean and standard deviation of the perineum length was 4.6+0.9 cm. The results showed that shorter perineum length (<4 cm) was associated with an increased risk of episiotomy, perineal tear, and instrumental delivery [17]. The results of our study are consistent with those of Rizk et al.

Aytun et al. studied severe perineal tear and the type of episiotomy in 400 primiparous women (2005). In their work, the rate of severe rupture was 2% (8 patients). In women with rupture, the perineal length was significantly shorter (P<0.001). The size of the baby's head circumference and birth weight were significantly larger and higher. In this study, the PB cutting point for perineal tears was 3.55 cm [19]. In the present study, 5.4% of women had a ruptured vaginal wall up to the sphincter or rectal mucosa, most of which were grade 1. In women with perineal tears, mean length PB, PB+GH, and AGD were significantly higher than those without tears. However, no significant difference was observed between the two groups in terms of the GH length, contradicting the findings of Aytun et al. In the present study, all cases of rupture occurred in women without episiotomy, indicating the effect of episiotomy in preventing perineal tear in women with shorter perineal length.

It seems that the delivery protocol without episiotomy is safe for the mother and infant [20]. In this context, drawing perineal sizes to determine the usefulness of episiotomy will be helpful [21]. Grouping women by perineal size, elective episiotomy is clinically feasible and effective. This policy seems to be associated with reducing perineal trauma associated with childbirth and is a useful tool for careful monitoring of delivery [22]. One of the limitations of this study is that it focused only on primiparous women, so the results cannot be generalized to multiparous women. However, the strength of our study was that, as only primiparous women were included (multiparous were not eligible), we controlled the potential bias of parity on perineal body length.

## CONCLUSION

In primiparous women with a gestational age of 37 to 42 weeks under vaginal delivery, shorter perineal size, vaginal hiatus, and anogenital distance significantly increased the likelihood of episiotomy and reduced the rate of first- and second-degree perineal tear. Therefore, perineal measurements could inform whether to perform an episiotomy.

## Personal thanks

All those who participated and assisted in implementing the design and collecting data are appreciated and thanked.
